# Nurse Managers' Toxic Leadership: Its Relation to Nurses' Internal and External Intentions to Whistleblowing

**DOI:** 10.1155/jonm/1734502

**Published:** 2025-06-28

**Authors:** Mahitab Mohamed Abdelrahman, Abdulqadir J. Nashwan, Doaa Fawzi El-Boudy

**Affiliations:** ^1^Nursing Administration and Education Department, College of Nursing, Prince Sattam Bin Abdulaziz University, Al-Kharj, Saudi Arabia; ^2^Nursing Administration Department, Faculty of Nursing, Suez Canal University, Ismailia, Egypt; ^3^Nursing and Midwifery Research Department, Hamad Medical Corporation, Doha, Qatar; ^4^Nursing Administration Department, Faculty of Nursing, Menofia University, Menofia, Egypt; ^5^Nursing Administration Department, Faculty of Nursing, Galala University, Suez, Egypt

**Keywords:** external whistleblowing, internal whistleblowing, nurses, toxic leadership, whistleblowing

## Abstract

**Aim:** This study aimed to explore the relationship between toxic leadership by nurse managers and staff nurses' intentions to engage in whistleblowing practices, both within and outside their hospital.

**Background:** Nurses are pivotal in providing high-quality care, but toxic nurse manager leadership in the workplace can lead to challenges in patient care and the workplace environment. In response, whistleblowing serves as a mechanism to mitigate these problems and foster accountability.

**Methods:** This descriptive correlational study was conducted from October 2023 to March 2024 with 292 inpatient and critical care unit nurses from a university hospital who were selected through simple random sampling. They completed the 15-item Toxic Leadership Scale to assess their views of toxic leadership in five domains: self-promotion, abusive supervision, unpredictability, narcissism, and authoritarian leadership. Nurses' intention to report misconduct was assessed using the 8-item whistleblowing intention questionnaire, including both internal and external whistleblowing. Descriptive statistics summarised the data; relationships between variables were evaluated using Spearman's correlation. Linear regression analysis provided the predictors of whistleblowing intention, exploring how personal characteristics and toxic leadership can affect this intention.

**Results:** The study revealed that nurses reported moderate levels of toxic leadership among their managers, with a total mean of 45.97 ± 11.545. They were more likely to intend to blow the whistle within the organisation (15.63 ± 3.085) than outside the organisation (10.77 ± 3.331), with the overall whistleblowing intention mean being 26.4 ± 5.008. Toxic leadership was significantly positively correlated with external whistleblowing (*r* = 0.282, *p* < 0.001).

**Conclusion:** A positive correlation was found between toxic leadership and external whistleblowing. Toxic leadership is a significant predictor of nurses' intentions to blow the whistle, and the educational level of nurses also contributes to these intentions. Addressing toxic leadership is crucial for encouraging whistleblowing and fostering a healthier work environment.

**Implications for Nursing Management:** Hospital managers and leaders need to combat toxic leadership, encourage a culture of safe whistleblowing practices, reinforce legal protections for whistleblowers and create ethical leadership training programs for nurse managers.

## 1. Introduction

Toxic leadership, a form of ineffective leadership, is becoming more common in the nursing sector. Toxicity negatively impacts the nursing staff's progress and creates a challenging work environment, job dissatisfaction, higher stress levels, increased turnover intentions, and poor outcomes for the nursing staff's commitment to the hospital [1]. A toxic leader can exhibit toxicity at various times, in different forms, and to varying degrees in similar settings. This impacts subordinates' perceptions of toxic leaders, either positively or negatively [2]. These perceptions are often guided by the behaviour of the leader, most often characterised by harassing, belittling, and intimidating employees—particularly followers. This behaviour creates unnecessary stress and pressure, reducing performance and causing other negative effects [3]. It is also characterised by maintaining control through harmful influencing tactics that, while often unintentional, cause serious harm due to reckless behaviour and incompetence [4].

Targeted initiatives within healthcare organisations can help alleviate the effects of toxic leadership behaviour. Studies have shown that nurse managers' transition programs, orientation, preceptorship, and mentorship programs are valuable in supporting nurses' transition into leadership roles. Through these programs, experienced nurse managers can provide organised mentorship, coaching, and feedback to nurses and junior nurse managers [5]. In addition, during the process of recruiting nurses for leadership positions, leadership assessment scales can be used to evaluate the emotional intelligence and leadership skills of the applicants. Further performance assessments, such as bottom-up or 360-degree feedback, can also be implemented to identify excellent leadership techniques [6, 7].

The connection between toxic leadership and whistleblowing in healthcare organisations is rooted in the impact of leadership behaviours on workplace culture and employee decision-making. Toxic leadership, characterised by abusive supervision, unpredictability, and authoritarianism, fosters an environment where fear and distrust prevail, making employees reluctant to engage in internal organisational reporting of misconduct or unsafe practices [7–9]. Research highlights that toxic leadership lowers work motivation and discourages whistleblowing due to fear or doubt [8, 10]. Whistleblowing is a necessary mechanism for addressing issues of safety and ethics, but its success ultimately requires transparency and support [11, 12]. Therefore, it is critical for organisations to foster ethical leadership practices and create safe avenues for whistleblowers. This ensures that employees do not feel like targets when raising concerns or reporting issues [8, 11].

Whistleblowing is the act of reporting violations of ethics or wrongdoings, especially withing educational and healthcare institutions [13, 14]. This action requires a great deal of moral courage to stand up for what is right, demand adherence to ethical standards, and be prepared to face the possible negative consequences of speaking up [13].

Nurses, in particular, are expected to raise concerns regarding any patient harm or impropriety [15]. In all organisations, internal whistleblowing is the recommended form of misconduct reporting. Many factors influence the practical implementation of internal whistleblowing within an organisation, including the size and structure of the organisation, leadership, policies and procedures, education and training, ethics, retaliation, social environment, organisational justice, reporting procedures, communication, and incentives [16]. If internal reporting policies fail to address the reported issues, nurses may seek external whistleblowing. Even at the institution's expense, when it prioritises patient interests, external whistleblowing can be morally justified. However, there is a tendency to avoid external reporting as it can adversely affect the healthcare organisation or service delivery [15].

Whistleblowing intentions are significantly influenced by two competing factors: the perceived severity of the wrongdoing and the anticipated risk of reprisal. Individuals are more likely to withhold disclosure when they perceive significant risks of retaliation. Conversely, the seriousness of the misconduct acts as a motivating factor, with highly intolerable violations increasing the likelihood of reporting due to heightened moral imperative and perceived ethical duty [17].

The growing awareness of whistleblower protection as a critical governance issue has prompted organisations to establish structured internal reporting mechanisms for suspected misconduct [18]. To foster trust in these systems, organisations must not only provide accessible reporting channels, such as in-house, outsourced, anonymous, or identified options, but also ensure procedural fairness by promptly and impartially investigating disclosures [16, 18]. In light of this, numerous public and private organisations have adopted measures and procedures to safeguard whistleblowers. In addition, regulatory bodies have formulated and introduced whistleblowing guidelines and policies [18].

Robust whistleblowing policies must ensure good-faith reporting, establish secure disclosure procedures, and mandate organisational support for whistleblowers. Transparency, confidentiality, and demonstrated interpersonal justice are integral to maintaining whistleblower confidence. Consequently, it is essential that independent third-party reporting pathways are available to preserve anonymity and mitigate fears of retaliation where internal processes lack impartiality [16].

Global healthcare systems are struggling with a high attrition rate of nursing staff, necessitating further research on factors influencing retention, including leadership approaches that enhance job satisfaction and reduce turnover [7]. While nursing research has established valuable insights into the context, process, and consequences of whistleblowing over the past two decades, critical theoretical gaps persist. There remain limited studies examining the conceptual foundations and application of whistleblowing within nursing practice [19]. Furthermore, although toxic leadership is increasingly recognised as a concern in nursing, existing literature does not thoroughly explain how toxic leadership disrupts work processes or examine the specific toxic behaviours exhibited by nurse managers [20]. This study aims to explore the relationship between toxic leadership by nurse managers and staff nurses' whistleblowing intentions, both within and outside their hospital. The objectives were to:

• Assess nurses' perceptions of toxic leadership among their managers.
• Evaluate staff nurses' intentions to report wrongdoing internally and externally.
• Explore how toxic leadership influences these reporting intentions.

Hypotheses Formulation:

  Hypothesis 1: Higher levels of toxic leadership behaviours by nurse managers negatively relate to nurses' intentions to report wrongdoing internally in the hospital.
  Employees can play a vital role in detecting misconduct in business and may blow the whistle to the relevant authorities within the organisation using different reporting channels. However, most employees remain silent due to fear of retaliation. Leaders can influence individuals' behaviour towards whistleblowing by creating safe avenues for reporting wrongdoing [21–23].
  This hypothesis is underpinned by evidence suggesting that toxic leadership behaviours (e.g., systematic destructive behaviours) erode trust and psychological safety in organisations and suppress internal whistleblowing [10, 24]. Toxic leadership has been associated with negative workplace outcomes such as absenteeism and deviant behaviours aligned with behaviours that would result in decreased engagement in organisational processes such as internal reporting [10, 25]. In contrast, nontoxic (ethical) leadership was positively linked to nurses' psychological safety and internal whistleblowing intentions [21].
  Hypothesis 2: Higher levels of toxic leadership behaviours exhibited by nurse managers are positively associated with staff nurses' intentions to report wrongdoing externally outside the hospital.

**Figure 1:**
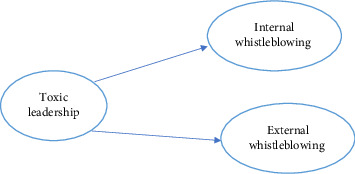
The conceptual framework of the study.

Studies show that toxic leadership produces a negative work environment, causing nurses to look externally for help with workplace problems. Toxic behaviours, specifically abuse and humiliation, further weaken trust in internal reporting mechanisms and compound dependence on external channels to provide accountability and protection [26, 27]. A study conducted by Mekawy and Ismail (2022) confirmed that nurses exposed to toxic leadership are also more likely to plan to quit or leave their organisations and exhibit higher turnover [27]. Moreover, studies show that toxic supervision makes employees more likely to engage in counterproductive work behaviours to retaliate against mistreatment [25]. These findings underscore the detrimental impact of toxic leadership on organisational health and the importance of establishing effective whistleblowing channels to address misconduct [10]. The conceptual framework for this study was developed, as illustrated in Figure 1, based on the literature review and the research hypotheses.

## 2. Methods

Research Design: This study used a descriptive correlational design according to Strengthening the Reporting of Observational Studies in Epidemiology (STROBE) guidelines to investigate toxic leadership and nurses' whistleblowing intentions. This design was selected because it captures prevailing patterns without manipulation, making it more suitable for evaluating toxic leadership influences within nursing practice.
Setting: This study was conducted within the inpatient and critical care units of a university (teaching) hospital. The hospital is crucial in medical education and healthcare delivery and has several medical specialities, from general medicine to surgery, paediatrics, obstetrics, gynaecology, and other specialised units. It provides healthcare for the local community and serves as a vital training centre for medical and nursing students, resident doctors and nurses, and other healthcare professionals.
Participants: The study involved 292 nurses from the university hospital, and Thompson's formula was used to estimate the sample size [28]. This equation was used with a population of 1200 nurses, a 95% confidence level (*z* = 1.96), a 5% margin of error (*d* = 0.05), and a 50% probability (*p* = 0.50). The questionnaire was given to 300 nurses, with eight voluntarily withdrawing from the study, resulting in a response rate of 97.33%.
  (1) $$\begin{array}{c} n=\frac{N\times p\;\left(1-p\right)}{\left[\left[N-1\times \left(d^{2}÷z^{2}\right)\right]+p\;\left(1-p\right)\right]}. \end{array}$$
A simple random sampling method was used to choose participants. The initial step was to compile a comprehensive list of nurses in each speciality. Each nurse was assigned a unique number. We used a random number generator to select each department's representative sample. This randomisation procedure guaranteed equal inclusion probability for all eligible nurses, thereby minimising selection bias and producing a representative sample.

### 2.1. Instruments

#### 2.1.1. Instrument I: Nurses' Personal Characteristics Datasheet

This instrument was developed to gather personal data from nurses such as age, marital status, sex, nursing education, years of work experience, and current department.

#### 2.1.2. Instrument II: Toxic Leadership Scale

The Toxic Leadership Scale was developed by Schmidt (2014) [29]. The researchers rigorously translated it into Arabic through linguistic and cultural validation processes. This scale assesses perceived toxic leadership behaviours in nurse managers with 15 items across five domains: self-promotion, abusive supervision, unpredictability, narcissism, and authoritarian leadership. Respondents rated each item on a 5-point Likert scale from 1 (“strongly disagree”) to 5 (“strongly agree”), with total scores ranging from 15 to 75, where higher scores reflect more pronounced toxic leadership behaviours.

The original version of the scale's psychometric validation showed good internal consistency: self-promotion (*α* = 0.85), abusive supervision (*α* = 0.79), unpredictability (*α* = 0.85), narcissism (*α* = 0.81), and authoritarian leadership (*α* = 0.84) [29]. This Arabic version has good overall reliability with Cronbach's *α* = 0.897 confirming the measurement properties of this scale in healthcare within a multicultural context.

#### 2.1.3. Instrument III: Whistleblowing Intention Questionnaire

This questionnaire was originally developed by Park and Blenkinsopp [30]. The questionnaire was translated into Arabic by the researchers to reflect the cultural and linguistic context of the study population. This scale assesses nurses' intentions to report misconduct internally (to their organisation) and externally (to outside regulatory bodies or authorities). The whistleblowing intention was measured using 8 items divided into internal and external whistleblowing, with the question, “If you found wrongdoing in your workplace, how hard would you try to do the following?” A five-point Likert scale with options ranging from “Not at all” (1) to “Very hard” (5). For each domain, total scores can be calculated independently, indicating the strength of nurses' intentions about internal or external reporting contexts. A higher score choice means more potential for whistleblowing behaviour.

The psychometric properties of the original tool suggest high reliability, with Cronbach's alpha being 0.878 for internal whistleblowing and 0.855 for external whistleblowing in the original [30]. The Arabic adaptation of the questionnaire showed high reliability for internal whistleblowing, with a Cronbach's alpha of 0.864, and for external whistleblowing, with a Cronbach's alpha of 0.917. The overall reliability was confirmed with a Cronbach's alpha of 0.846, indicating strong reliability.

### 2.2. Validity of Instruments

To validate the instruments, the researchers initially translated the tools into Arabic. Subsequently, bilingual experts back-translated the resulting versions to determine whether the translated items accurately captured the original English versions. Five bilingual experts were asked to evaluate the translated instruments in various aspects, including relevance, clarity, completeness, and potential bias. Based on their feedback, adjustments were made to improve the translations and ensure the final versions were valid. The data were analysed using statistical software implementing Cohen's Kappa coefficient to assess inter-rater agreement. It showed significant agreement above 0.80, which indicated nearly perfect agreement beyond chance. This result illustrates the evaluations' high inter-rater reliability and consistency [31]. The updated instruments were tested for reliability in a pilot study before they were implemented in the main study.

### 2.3. Data Collection

Data collection occurred from October 2023 to March 2024. A pilot study was conducted with 30 nurses (10% of the sample) to evaluate clarity, feasibility, and time to complete the instruments. The instruments were well understood, and the average time for completion was less than 15 min. As no considerable issues were identified, the pilot sample was included in the final study. Collaboration with head nurses ensured smooth data collection. Meetings with participants were held twice a week across various shifts. Nurses were provided with a description of the study's aim and objectives, basic instructions about the research instruments, and envelopes for informed consent. To facilitate participation, the researcher met with nurses during breaks to explain the study's purpose. Questionnaires were then administered for self-completion using validated instruments to improve reliability. Clear instructions were given to minimise misinterpretation. The researcher left participants to fill out the forms independently and later collected the responses. Data were treated with strict confidentiality and anonymity. Participants were informed that participation was voluntary and they could withdraw from the study at any time. Ethical clearance was granted according to bioethical standards.

### 2.4. Data Analysis

SPSS 26.0 (IBM Inc., Chicago, IL, USA) was used for data analysis. Participants' personal information was presented as frequencies and percentages. Descriptive statistics, including mean and standard deviation, were applied to describe the toxic leadership scale, and whistleblowing intention questionnaire. Data homogeneity was assessed using the Kolmogorov–Smirnov test. Spearman's nonparametric correlation identified associations between variables, with a significance level set at *p* < 0.05 and a 95% confidence interval. The Mann–Whitney and Kruskal–Wallis tests were used to determine differences between study groups. Linear regression identified factors predicting whistleblowing intention, including toxic leadership and demographic characteristics.

### 2.5. Ethical Considerations

The research was approved by the Ethics Committee of the Faculty of Nursing at Menofia University (Approval No. 1013). The study followed the principles of the Declaration of Helsinki. Participants were fully informed about the research's purpose, methods, and voluntary nature. Informed consent was obtained, with participants assured of their right to withdraw from the study at any time without consequences. All data were anonymised, securely stored, and accessible only by the research team, ensuring confidentiality and anonymity. Ethical considerations were upheld during data collection. Interviews were scheduled during nurses' breaks to respect their time and work schedules, promoting honest responses.

## 3. Results

In Table 1, the study participants had a mean age of 27.92 ± 4.305 years. Most were female (76.370%), married (60.274%), and held a technical nursing degree (59.932%). The nurses' work experience averaged 6.13 ± 3.574 years, and 55.137% worked in noncritical areas.

As shown in Table 2, no significant relationship was found between participants' toxic leadership and their personal characteristics, except for their education (*p* < 0.001). A significant relationship existed only between participants' whistleblowing intention and their education (*p* < 0.001).

Table 3 shows that the highest mean domain is authoritarian, with a mean score of 9.63, followed by self-promotion, with a mean score of 9.51. The participants' managers demonstrated moderate levels of toxic leadership, with a mean score of 45.97. Nurses showed a greater inclination toward internal whistleblowing (mean score of 15.63) than external whistleblowing (mean score of 10.77).

Table 4 demonstrates a significant correlation between external whistleblowing and toxic leadership (*r* = 0.282, *p* < 0.001). A significant correlation was also found between overall whistleblowing and toxic leadership (*r* = 0.228, *p* < 0.001).

Table 5 presents the results of a linear regression model conducted to predict whistleblowing among participants. The dependent variable, whistleblowing, is regressed on various predictor variables, including toxic leadership (*B* = 0.248, *t* = 4.499, *p* < 0.001) and their education (*B* = 0.300, *t* = 5.205, *p* < 0.001).

## 4. Discussion

Whistleblowing among nurses represents a critical mechanism for exposing and addressing malpractice, ethical violations, and potential hazards within healthcare systems. This courageous act is significantly influenced by organisational leadership, as leaders play a pivotal role in shaping the behaviour of subordinates. The literature suggests that ethical leaders can foster a culture that encourages and supports whistleblowing [32]. Conversely, toxic leadership may create barriers to reporting misconduct [33]. The current study specifically investigates the relationship between nurse managers' toxic leadership characteristics and staff nurses' whistleblowing intentions, addressing an important gap in understanding how different leadership styles influence reporting misbehaviours in healthcare settings.

### 4.1. Staff Nurses' Perceptions of Nurse Managers' Toxic Leadership

The findings reveal that nurses perceive their leadership as moderately toxic, with three dominant traits emerging: authoritarianism, self-promotion, and narcissism. These results are consistent with previous studies finding moderately toxic leadership patterns manifesting among nurse managers [7, 34]. Authoritarian behaviours manifest through unilateral decision-making and management styles that exclude staff input. Self-promotion happens when leaders focus excessively on their personal achievements rather than team accomplishments, while narcissistic tendencies appear in self-absorbed behaviours [34].

Nursing leaders often decide independently without involving their nursing staff. Consequently, the former are perceived as controlling and directive instead of approachable and inclusive [35]. Notably, teaching hospital nurses view their leaders as having lower toxic leadership compared to their counterparts in general hospitals, suggesting that structured leadership training at the teaching hospital may help mitigate toxic behaviours and foster positive organisational relationships [35, 36]. However, even minimally perceived toxic behaviours can negatively impact nursing retention and organisational climate, as Hossny et al. [37] found. This paradox suggests that nurses may normalise certain destructive behaviours as inherent to healthcare leadership [37]. Mahgob et al. also reported that minimal toxic leadership has negative effects on nursing staff as work engagement levels remained moderate and showed a negative correlation with toxic leadership [36].

The literature has consistently recommended that leadership training interventions can yield measurable improvements in both leader behaviours and staff outcomes, emphasising the need for systematic leadership development programs in healthcare settings [35–37].

### 4.2. Staff Nurses' Intentions to Whistleblow

This study demonstrates that nurses' educational background significantly influences their whistleblowing intention. This relationship is well-supported in the literature, which identifies ethics training and advanced healthcare qualifications as key factors that cultivate the moral courage necessary for whistleblowing [38–40]. Moral courage plays a pivotal role in fostering a whistleblowing culture by empowering employees to speak up despite workplace political risks or potential negative consequences [41].

The study also reveals nurses' strong preference for internal whistleblowing channels within their organisation. This is mainly driven by organisational loyalty and fear of damaging organisation's reputation by external whistleblowing. This reflects nurses' dual commitment to patient safety and institutional integrity, influenced by significant deterrents including fear of retaliation, professional repercussions, and skepticism about external reporting efficacy [12, 15, 17, 42].

The findings corroborate existing literature documenting nurses' positive attitudes toward whistleblowing as an ethical obligation [43]. Sachdeva et al. have also found that nurses considered internal reporting morally fulfilling [42]. Nevertheless, while nurses derive moral satisfaction from internal reporting, persistent fears of job loss and workplace harassment create substantial behavioural constraints which may hinder whistleblowing [42]. Chaudhary et al. demonstrated that ineffective internal channels and organisational indifference serve as critical tipping points, pushing employees toward external disclosure when internal processes prove unresponsive, the wrongdoing is serious, and/or the moral cost of nonreporting becomes untenable [44].

### 4.3. The Relationship Between Nurse Managers' Toxic Leadership and the Nurses' Intention to Initiate Whistleblowing

The current study found a significant positive correlation between external whistleblowing and toxic leadership. This indicates that higher levels of toxic leadership are associated with an increased likelihood of individuals reporting misconduct externally. Toxic leadership can create a hostile work environment, leading nurses to report unethical or harmful practices to external authorities to address the issues more effectively and safeguard both their patients as well as nurses' professional integrity. In this context, Labrague found that nurses working under a manager with toxic behaviours experienced more adverse events [1]. In contrast, ethical leadership increases nurses' willingness to voice concerns through internal channels and enhances their freedom to speak [21].

The literature also reports that individuals are more likely to report wrongdoing when they perceive it as serious but are reluctant to do so if they anticipate major retaliation. This helps explain why nurses might choose external whistleblowing when faced with toxic leadership. Seeing the issue as serious motivates reporting, but at the same time, fear of retaliation influences their choice of how to report. This also shows how people rationalise the situation regarding both the severity of the transgression and the motivation to disclose it [17].

Nurse managers must cautiously approach whistleblowing situations since their handling of confidentiality and concerns pose a significant impact, not only on the people involved, but equally on the organisation. Understanding and applying confidentiality can be critical to the outcomes of whistleblowing within healthcare organisations since it can be a double-edged sword that shields organisations while leaving whistleblowers feeling isolated [45].

### 4.4. Implications for Nursing Management

Our findings offer critical insights for healthcare policymakers and organizations. Hospitals should implement whistleblower protection systems and promote ethical leadership, to cultivate a culture of transparency and accountability. These systems should include confidential reporting channels that guarantee anonymity, incentive programs to recognise and promote ethical reporting behaviours, and accessible legal counsel. Legal guidance should be made available to whistleblowers to ensure they understand their rights and protections, reducing fear of retaliation and promoting whistleblowing. Such measures not only safeguard organizational integrity, accountability, and safety but also encourage internal whistleblowing while reducing the need for external disclosures.

Ethical leadership genuinely protects whistleblowers leading to better risk management and less misconduct. Therefore, healthcare institutions should incorporate ethical leadership development programs. These programs should focus on building emotional intelligence, conflict resolution skills, and ethical decision-making capabilities. Regular leadership evaluations such as 360-degree feedback systems can help identify and address toxic behaviours proactively, preventing the escalation of harmful leadership practices. Concurrently, regular training sessions should be conducted to educate nurses and managers about whistleblowing protocols including both internal and external reporting mechanisms.

When nurses perceive management as unsupportive or inaccessible, workplace environments quickly become toxic rather than collaborative. To prevent this, organizations should establish clear reporting guidelines and provide resources that empower staff to voice concerns effectively. Creating a structured confidential mechanism specifically for reporting leadership misconduct would also help combating the negative impact of toxic leaders.

The importance of open communication channels cannot be overstated. Collaboration workshops can enhance nurses' and managers' decision-making, teamwork, and communication skills. Nurse managers also require specialised training in whistleblowing policies to ensure proper handling of investigations while maintaining confidentiality and transparency.

Looking forward, research should explore how cultural and organisational factors influence whistleblowing behaviours across healthcare settings. Cross-cultural studies comparing collectivist versus individualist work environments could elucidate variations in reporting tendencies, while also examining how work environments impact nurses' propensity to report improper conduct. Longitudinal research or experimental designs may provide deeper insight into the potential lasting effects of toxic leadership on whistleblowing practices and establish causal links between these variables over time.

### 4.5. Limitations

This study recognises the limitations of self-reported measures, including social desirability and recall bias. To address these risks, participant anonymity and confidentiality were assured to promote truthful responses. Nevertheless, relying on self-reported data remains a significant limitation, whether through bias with participants' perceptions, inaccuracies in recall, or how people tend to report socially acceptable answers. Future studies could attempt to overcome this by augmenting the data collection with other types of information, such as reports and records on whistleblowing events, to triangulate evidence and improve accuracy. Another limitation of this study is that it took place at a single university hospital, which may affect the generalizability of our findings in other healthcare settings. Involving multiple hospitals from different healthcare facilities (e.g., private, public, and specialised) increases the external validity and generalizability of the results to more diverse healthcare systems.

## 5. Conclusion

The study found that nursing managers displayed moderate levels of toxic leadership behaviours. While nursing professionals generally preferred to report concerns internally, the research uncovered that under toxic leadership, nurses were significantly more likely to bypass internal reporting channels and escalate concerns to external authorities. This novel finding suggests that external whistleblowing may serve as a last resort when internal systems are compromised by toxic management.

In the regression analysis, toxic leadership emerged as a significant predictor of whistleblowing intentions, with higher levels of toxic leadership associated with an increased likelihood of whistleblowing behaviour. Nursing education level also served as a contributing factor, likely due to enhanced ethical training and professional confidence associated with advanced qualifications. These findings fill a critical gap in the literature, establishing toxic leadership as a unique driver of external whistleblowing in healthcare settings, a relationship previously undocumented in nursing research.

This study highlights the critical need for healthcare organizations to address toxic leadership and strengthen internal reporting systems, thereby creating environments where nurses can safely voice concerns through appropriate internal channels while preserving organizational integrity and patient safety. Future research should explore targeted interventions to mitigate toxic leadership's impact and evaluate their effectiveness in promoting ethical whistleblowing practices.

## Figures and Tables

**Table 1:** Personal characteristics of the study participant (*N* = 292).

Personal characteristics	Category	No.	(%)
Age	20–< 30 years	213	72.945
	30–< 40 years	71	24.315
	≥ 40 years	8	2.740
	Mean ± SD	27.92 ± 4.305	

Marital status	Unmarried	116	39.726
	Married	176	60.274

Sex	Female	223	76.370
	Male	69	23.630

Nursing education	Diploma	95	32.534
	Associate (technical)	175	59.932
	Bachelor	22	7.534

Years of experience	≤ 5 years	136	46.575
	> 5 years	156	53.425
	Mean ± SD	6.13 ± 3.574	

Department	Noncritical departments	161	55.137
	Critical departments	131	44.863

**Table 2:** Relation between study variables and personal characteristics of the study participants (*n* = 292).

Personal characteristics	Category	Toxic leadership			Whistleblowing intention		
		Mean (S.D)	H/U	*p* value	Mean (S.D)	H/U	*p* value
Age (years)^a^	20–< 30 years	45.78 ± 11.590	0.209	0.901	26.690 ± 5.031	3.254	0.196
	30–< 40 years	46.39 ± 11.753			25.563 ± 4.956		
	≥ 40 years	47.25 ± 9.301			26.375 ± 4.565		

Gender^b^	Female	45.74 ± 11.532	8132.0	0.474	26.381 ± 4.660	7733.0	0.948
	Male	46.72 ± 11.639			26.492 ± 6.035		

Marital status^b^	Unmarried	46.24 ± 11.520	10,238.5	0.966	26.853 ± 5.123	9261.50	0.178
	Married	45.80 ± 11.591			26.113 ± 4.924		

Nursing education^a^	Diploma	45.15 ± 10.241	15.709	< 0.001^∗^	25.484 ± 5.084	51.059	< 0.001^∗^
	Associate (technical)	45.03 ± 11.320			25.880 ± 4.140		
	Bachelor	57.05 ± 13.30			34.590 ± 3.686		

Experience (years)^b^	≤ 5 years	45.76 ± 11.284	10,790.0	00.800	26.404 ± 5.856	10,279.0	0.646
	> 5 years	46.15 ± 11.801			26.410 ± 4.150		

Department^b^	Critical departments	46.33 ± 12.379	10,251.0	0.681	26.427 ± 4.617	10,746.0	0.779
	Noncritical departments	45.68 ± 10.850			26.391 ± 5.319		

^a^Kruskal–Wallis test (*H*-test).

^b^Mann–Whitney test (*U*-test).

^∗^ *p* < 0.05.

**Table 3:** Descriptive statistics of nurse managers' toxic leadership and whistleblowing domains as perceived by nurses (*N* = 292).

Domains	Minimum	Maximum	Mean	SD
Self-promotion	3	15	9.51	2.862
Abusive supervision	3	15	8.61	2.951
Unpredictability	3	15	8.74	2.726
Narcissism	3	15	9.48	2.771
Authoritarian leadership	3	15	9.63	2.500
Toxic leadership	15	75	45.97	11.545
External whistleblowing (EWB)	4	20	10.77	3.331
Internal whistleblowing (IWB)	4	20	15.63	3.085
Total whistleblowing	8.00	40.00	26.4075	5.008

**Table 4:** Correlations among the study variables (*N* = 292).

Variables		1	2	3	4
Toxic leadership (1)	*r*	1			
	*p*	—			

Total whistleblowing (2)	*r*	0.228^∗∗^	1		
	*p*	< 0.001	—		

External whistleblowing (3)	*r*	0.282^∗∗^	0.758^∗∗^	1	
	*p*	< 0.001	< 0.001	—	

Internal whistleblowing (4)	*r*	0.073	0.673^∗∗^	0.097	1
	*p*	0.215	< 0.001	0.099	—

^∗∗^Correlation is highly significant at *p* < 0.01.

**Table 5:** Regression model of the predictors' factors for whistleblowing among participants (*n* = 292).

Model	Unstandardised coefficients		Standardised coefficients	*t*	Sig.	95.0% confidence interval for B	
	*B*	Std. Error	Beta			Lower bound	Upper bound
(Constant)	19.922	3.337		5.969	< 0.001	13.352	26.491
Toxic leadership	0.108	0.024	0.248	4.499	< 0.001	0.061	0.155
Age	−0.078	0.137	−0.067	−0.572	0.568	−0.347	0.191
Marital status	−0.675	0.589	−0.066	−1.146	0.253	−1.835	0.485
Sex	−0.005	0.671	0.000	−0.007	0.995	−1.326	1.317
Nursing education	2.583	0.496	0.300	5.205	< 0.001	1.606	3.559
Years of experience	0.231	0.126	0.164	1.830	0.068	−0.018	0.480
Department	0.070	0.564	0.007	0.123	0.902	−1.040	1.179

*Note: R* = 0.421; *R*^2^ = 0.177; Adjusted *R*^2^ = 0.151; *F* = 6.755.

^∗^ *p* < 0.05.

## Data Availability

The data that support the findings of this study are available from the corresponding authors upon reasonable request.
